# Supplementary material for: A Nutritionally Complete Oral Nutritional Supplement Powder Improved Nutritional Outcomes in Free-Living Adults at Risk of Malnutrition: A Randomized Controlled Trial
Source: Int J Environ Res Public Health. 2022 Sep 9;19(18):11354. doi: 10.3390/ijerph191811354 (PMC9517313; doi:10.3390/ijerph191811354)
Supplement: Supplementary file 1 [file ijerph-19-11354-s001.zip › ijerph-1870354-supplementary.pdf]

Supplementary Table S1. Nutrient composition of study product.

Nutrient	per 100 g Powder	per Serving (4.5 Scoops/69 g Powder)
Energy, kcal	435	300
Fat, g	14.5	10.0
of which saturated fatty acids, g	1.19	0.82
of which monounsaturated fatty acids, g	9.54	6.58
of which polyunsaturated fatty acids, g	3.74	2.58
Carbohydrate, g	57.7	39.8
of which sugars, g	16.6	11.5
of which lactose, g	≤ 0.87	≤ 0.60
Fibre, g	4.35	3.00
Protein, g	16.2	11.2
Sodium, mg	304	210
Chloride, mg	464	320
Potassium, mg	652	450
Calcium, mg	290	200
Phosphorus, mg	203	140
Magnesium, mg	101	70
Iron, mg	6.7	4.6
Zinc, mg	4.9	3.4
Copper, µg	696	480
Iodine, µg	61	42
Selenium, µg	31	21
Manganese, mg	1.57	1.08
Chromium, µg	31	21
Molybdenum, µg	41	28
Fluoride, mg	0.58	0.40
Vitamin A, µg RE	401	277
of which ß-Carotene, µg RE	97	67
Vitamin D3, µg	5.8	4.0

Vitamin E, mg α -TE	7.8	5.4
Vitamin K1, μg	28	19
Vitamin C, mg	57	39
Thiamin (vitamin B1), mg	0.65	0.45
Riboflavin (vitamin B2), mg	0.70	0.48
Vitamin B6, mg	0.8	0.6
Niacin, mg/mg NE	7.5/10.3	5.2/7.1
Folic Acid, μg	61	42
Vitamin B12, μg	1.48	1.02
Pantothenic Acid, mg	2.6	1.8
Biotin, μg	19.6	13.5
Choline, mg	159	110

RE, retinol equivalents; α -TE, alpha-tocopherol equivalents; NE, niacin equivalents.

Supplementary Table S2. Micronutrient intakes at baseline, change from baseline and the estimated mean group difference in change from baseline between the intervention group (*n* = 42) and control group (*n* = 40).

	Group	Mean ± SD	Change from Baseline, Mean ± SD	Between group Difference in Change from Baseline	
		**Baseline**	**12 weeks**	**Estimated Mean (95% CI)[a]**	**P-value**
Vitamin A, RE	Intervention	889 ± 882	551 ± 1319	**645 (93, 1198)**	**0.023**
	Control	1046 ± 996	-94 ± 1186		
Thiamin, mg	Intervention	1.16 ± 0.47	0.58 ± 0.56	**0.61 (0.38, 0.84)**	**<0.001**
	Control	1.16 ± 0.51	-0.03 ± 0.46		
Riboflavin, mg	Intervention	1.27 ± 0.41	0.62 ± 0.48	**0.61 (0.40, 0.82)**	**<0.001**
	Control	1.32 ± 0.44	0.01 ± 0.45		
Niacin, mg NE	Intervention	23.6 ± 9.2	10.5 ± 10.5	**8.8 (4.2, 13.5)**	**<0.001**
	Control	23.7 ± 10.6	1.7 ± 10.6		
Vitamin B6, mg	Intervention	1.7 ± 0.6	0.7 ± 0.1	**0.7 (0.5, 1.0)**	**<0.001**
	Control	1.7 ± 0.7	-0.1 ± 0.5		
Vitamin B12, μg	Intervention	2.98 ± 2.37	2.36 ± 3.57	**2.43 (1.01, 3.85)**	**0.001**
	Control	3.61 ± 2.19	-0.07 ± 2.81		
Biotin, μg	Intervention	16.8 ± 10.5	22.5 ± 10.1	**22.0 (18.2, 25.8)**	**<0.001**
	Control	16.7 ± 7.2	0.5 ± 7.1		
Vitamin C, mg	Intervention	112 ± 72	54 ± 78	**51 (20, 83)**	**0.002**
	Control	111 ± 67	3 ± 66		
Vitamin E, mg α -TE	Intervention	5.9 ± 2.6	9.9 ± 3.0	**9.5 (8.2, 10.8)**	**<0.001**
	Control	6.4 ± 3.2	0.3 ± 3.0		
Folate, μg	Intervention	246 ± 228	43 ± 244	48 (-33, 129)	0.241
	Control	218 ± 102	-5 ± 83		

Vitamin K, µg	Intervention	60 ± 78	35 ± 78	14 (-25, 52)	0.475
	Control	48 ± 46	21 ± 97		
Pantothenic acid, mg	Intervention	4.0 ± 1.5	2.5 ± 1.7	**2.4 (1.7, 3.1)**	**<0.001**
	Control	3.9 ± 1.4	0.1 ± 1.3		
Chloride, mg	Intervention	267 ± 182	502 ± 187	**450 (354, 547)**	**<0.001**
	Control	298 ± 171	52 ± 248		
Copper, µg	Intervention	1131 ± 574	711 ± 608	**653 (437, 869)**	**<0.001**
	Control	1061 ± 393	58 ± 328		
Iodine, µg	Intervention	47 ± 28	68 ± 45	**66 (48, 85)**	**<0.001**
	Control	51 ± 28	2 ± 39		
Iron, mg	Intervention	11.5 ± 5.1	6.5 ± 5.5	**6.7 (4.6, 8.8)**	**<0.001**
	Control	11.4 ± 4.9	-0.3 ± 3.9		
Magnesium, mg	Intervention	271 ± 104	81 ± 107	**92 (53, 132)**	**<0.001**
	Control	275 ± 99	-12 ± 69		
Manganese, mg	Intervention	3.46 ± 1.83	1.22 ± 1.93	**1.31 (0.54, 2.09)**	**0.001**
	Control	3.10 ± 1.72	-0.10 ± 1.57		
Molybdenum, µg	Intervention	15 ± 14	48 ± 20	**48 (41, 55)**	**<0.001**
	Control	14 ± 9	0 ± 10		
Phosphorus, mg	Intervention	1000 ± 316	127 ± 333	129 (-7, 265)	0.064
	Control	1030 ± 338	-1 ± 283		
Potassium, mg	Intervention	2147 ± 821	536 ± 813	**595 (264, 927)**	**0.001**
	Control	2293 ± 829	-60 ± 688		
Selenium, µg	Intervention	98 ± 35	33 ± 39	**26 (9, 43)**	**0.004**
	Control	99 ± 43	7 ± 39		
Sodium, mg	Intervention	3033 ± 983	420 ± 1548	409 (-292, 1109)	0.249
	Control	3297 ± 1023	12 ± 1640		

Zinc, mg	Intervention	8.0 ± 3.0	5.3 ± 3.7	**5.4 (3.9, 6.9)**	**<0.001**
	Control	8.1 ± 2.8	-0.1 ± 3.1		

RE, retinol equivalents; α -TE, alpha-tocopherol equivalents; NE, niacin equivalents; CI, confidence interval. Data are presented as mean ± SD. Bold values represented $p < 0.05$. [a] Represents group difference (intervention group minus control group) in 12-week change estimate (12-week minus baseline). Estimated mean (95% CI) was derived from independent sample t-test.
